# Sexual Self-Concept in Fertile and Infertile Women: A Comparative Study

**DOI:** 10.22074/IJFS.2021.6205

**Published:** 2021-01-27

**Authors:** Hajar Lotfollahi, Hedyeh Riazi, Reza Omani-Samani, Saman Maroufizadeh, Ali Montazeri

**Affiliations:** 1 Students Research Office, School of Nursing and Midwifery, Shahid Beheshti University of Medical Sciences, Tehran, Iran; 2 Department of Midwifery and Reproductive Health, School of Nursing and Midwifery, Shahid Beheshti University of Medical Sciences, Tehran, Iran; 3 Department of Medical Ethics and Law, Reproductive Biomedicine Research Center, Royan Institute for Reproductive Biomedicine, ACECR, Tehran, Iran; 4 Department of Biostatistics, School of Nursing and Midwifery, Guilan University of Medical Sciences, Rasht, Iran; 5 Population Health Research Group, Health Metrics Research Center, Iranian Institute for Health Sciences Research, ACECR, Tehran, Iran

**Keywords:** Fertility, Infertility, Self-Concept, Sexual Health

## Abstract

**Background:**

Sexual self-concept has a considerable impact on mental and sexual health. However, the relationship between sexual self-concept and infertility is unknown. This study aimed to compare sexual self-concept between fertile and infertile women.

**Materials and Methods:**

This cross-sectional study was conducted on a sample of 250 fertile and 250 infertile women who had referred to 9 health centers affiliated to Medical universities in Tehran and Royan infertility treatment clinics in Tehran, Iran in 2017. Sexual self-concept was measured using the Multidimensional Sexual Self-Concept Questionnaire (MSSCQ) consisting of 20 subscales. Analysis of covariance (ANCOVA) was performed to compare sexual self-concept between the two groups.

**Results:**

The mean age of fertile and infertile women was 34 ± 5.62 and 29.74 ± 5.29 years, respectively. The high- est score in both groups was for the sexual self-schemata subscale (mean score for fertile=3.21 ± 0.68 and for infer- tile=3.42 ± 0.62). The lowest score was for sexual-depression subscale (mean score for fertile=0.59 ± 0.81 and for infertile=0.61 ± 0.76). After adjustment for the age of each subject, the husband's age, duration of marriage, and wom- en’s education, we analyzed the sexual-satisfaction, the power-other sexual control, and the fear-of-sex subscales, which were found to be significantly lower in infertile women (P<0.05). No other significant differences between the fertile and infertile groups were observed.

**Conclusion:**

We observed significant differences between fertile and infertile women in terms of sexual-satisfaction, the power-other sexual control, and the fear-of-sex, but not in other sexual self-concept subscales. These findings sug- gest that there is need to improve sexual self-concept among both fertile and infertile women. Indeed implementation of educational and counseling programs by reproductive health specialists might play an important role in enhancing sexual self-concept among these populations.

## Introduction

Sexual health is a broad concept that contains many dimensions including sexual self-concept (1). Sexual selfconcept refers to the totality of an individual as a sexual being, which includes positive and negative believes and emotions (2). In fact sexual self-concept refers to the feelings, concepts, and behaviors about sexual relationships (3). Thus, sexual self-concept plays an important role in sexual behavior and reproductive health (4), and is a key indicator of successful sexual activity (5). Indeed sexual self-concept might influence sexual life in many ways both in men and women.

Although studies on sexual self-concept are limited, existing evidence suggests that negative sexual self-concept could be associated with lower sexual activity, low sexual satisfaction, fear of sex, and other sexual-related psycho-logical problems. For instance, a recent review on factors affecting sexual self-concept indicated that age, gender, sexual transmitted infections, body image, sexual abuse in childhood, and mental health might be associated with sexual self-concept (6). A study among a sample of postmenopausal women found that there were a significant association between sexual self-concept and stress, anxiety and depression (5).

A pile of evidence exists on sexual health in infertile couples, but little is known about sexual self-concept and infertility. It is argued that since infertility by itself imposes several emotional and psychological problems, the addition of impaired sexual self-concept among infertile couples could deepen the problem even further (7, 8). In fact, sexual self-concept as one of the prominent indicators of sexual function is expected to be highly affected in infertile women (9) and thus merits investigation. Additionally, since in some societies such as Iran women are more vulnerable to infertility problems than other countries (10), it seems that studying sexual self-concept in infertile women is of major importance. Infertility brings negative feelings for women such as guilt and shame. They usually feel that their integrity as a woman is compromised and cannot play their feminine role as expected. They are worried that their marital life will be at risk. Generally, men will cope more easily with infertility and are less emotionally affected by it compared to women (11). However, to our best knowledge, no study exists to address sexual self-concept and infertility. Thus, the present study aimed to assess sexual self-concept among a sample of fertile and infertile women. The findings of this study could contribute to the existing literature regarding sexual health and may help improve related policies and practices.

## Materials and Methods

### Design and participants

This cross-sectional study was conducted on a sample of fertile and infertile women in 2017. The infertile women were recruited from the Royan Institute by a convenience method while the fertile women were recruited from health centers affiliated to medical universities in Tehran, Iran (Iran University of Medical Sciences, Shahid Beheshti University of Medical Sciences, and Tehran University of Medical Sciences). A list of appropriate health centers was provided and based on the required sample size, preferably from different geographical areas, three centers affiliated to each university were selected (a total of 9 centers). The centers affiliated to each university were coded and a colleague who was not connected to the study randomly drew three centers. Then, proportional to the population a convenience sample of fertile women from each center was entered into the study. The required sample size for the study was estimated based on the smallest mean score difference between the two groups derived from a pilot study. We estimated that 234 women are needed for each group in order to be able to detect at least 0.15 mean score difference for sexual self-concept between the two groups considering a standard deviation of 0.82 with 80% statistical power at 5% significance level. However, allowing for a 10% drop out a sample of 250 was chosen for each group using the following formula:

n=(zα2+zβ)2σ2(µ1-µ2)2

n=(1.96+0.84)2×0.822(1.60-1.45)2

n=7.84×0.670.0225=234

Inclusion criteria for fertile women were: having at least one healthy child, using a contraceptive method, not having a nursing infant and no history of infertility. The infertile women were included if they had a primary infertility problem. However, the inclusion criteria for all participants were: being of Iranian origin, aged between 18-45 years, and with no medical or psychological disorders. In addition, they would not have any records of alcohol or drug abuse and would not have to be taking libido effective drugs.

### Measures

The data were collected using a short questionnaire containing items on demographic information and the multidimensional Snell's Sexual Self-Concept Questionnaire (MSSCQ). The questionnaire was designed and validated by Snell (12). The MSSCQ is a self-report instrument, measuring the various dimensions of sexual self-concept with 100 items and 20 subscales. The questionnaire measures both positive and negative concepts. The positive concepts refer to those such as sexual motivation, sexual satisfaction and sexual schemata. For instance, sexual schemata is defined as a cognitive framework that organizes and guides the processing of information about the sexual-related aspects of oneself. The negative concepts refer to concepts such as sexual anxiety, sexual depression, and power-other sexual control. The later subscale (power-other sexual control) is defined as the belief that the sexual aspects of one's life are controlled by others who are more powerful and influential than oneself. The detailed descriptions of the subscales are explained by Snell (12). Each subscale contains 5 items and is rated on a five-point Likert scale with scores ranging from 0 to 4. The mean score for each subscale is considered as the cutoff point and a score above the mean indicates a higher tendency for that subscale. The psychometric properties of the questionnaire are described previously. The Cronbach's alpha coefficient for the questionnaire was reported to be between 0.72 and 0.94. Reliability and validity of the Persian version of the questionnaire are well documented in previous studies. The Cronbach's alpha coefficient for the questionnaire varies from 0.41 to 0.87 (13, 14).

### Statistical analysis 

Statistical analysis was performed using the IBM SPSS Statistics for Windows, Version 22.0 (IBM Corp., Armonk, NY, USA). The continuous variables were expressed as the mean and standard deviation (SD), and the categorical variables were expressed as numbers and percentages. When appropriate, Chi-square test and independent t test were used to compare baseline characteristics between fertile and infertile women. Analysis of Covariance (ANCOVA) was used to compare sexual selfconcept between groups while adjusting for the subject’s age, her husband’s age, duration of their marriage, and her level of education. All statistical tests were two-tailed and the level of significance was set at P≤0.05.

### Ethical considerations

The Ethics Committee of Shahid Beheshti University of Medical Sciences, Tehran, Iran, approved this study (IR.SBMU.PHNM.1394.263). The participants were informed of the purpose of the study and were assured of confidentiality. After signing a consent form and agreeing to participate, all women completed the questionnaires.

## Results

The mean age of fertile and infertile women was 34 ± 5.62 and 29.74 ± 5.29 years, respectively. The educational level of most fertile and infertile women and their husbands was at secondary and university levels. In both groups, the majority of women were housewives and the economic status of households was moderate. The mean duration of infertility was 5.54 ± 3.868 years. The demographic characteristics of the participants are presented in Table 1.

**Table 1:** Distribution of some demographic variables in fertile and infertile women


Variables	Fertile women n=250	Infertile women n=250	P value

Age (Y)	34 ± 5.62	29.74 ± 5.29	<0.001^*^
Husband’s age (Y)	38.88 ± 6.41	33.99 ± 5.29	<0.001^*^
Duration of marriage (Y)	12.46 ± 5.78	7.12 ± 3.91	<0.001^*^
Education			0.001^*^^*^
Elementary	28 (11.2)	37 (14.8)	
Secondary	132 (52.8)	91 (36.4)	
University	90 (36)	122 (48.8)	
Job			0.740^*^^*^
Housewife	200 (80)	197 (78.8)	
Employed	50 (20)	53 (21.2)	
Economic status			0.213^*^^*^
Weak	30 (12)	39 (15.6)	
Moderate	176 (70.4)	179 (71.6)	
Good	44 (17.6)	32 (12.8)	


Values are given as mean ± SD or n (%). *; Derived from independent t test and **; Derived from Chi-square test.

The highest and the lowest mean scores for sexual selfconcept in both groups were for the sexual self-schemata (mean score=3.21 ± 0.68 and 3.42 ± 0.62) and the sexualdepression subscales (mean score=0.59 ± 0.81 and 0.61 ± 0.76), respectively, indicating that both groups were well prepared for processing of information about the sexualrelated aspects of oneself and similarly had a low level of sexual related depression. Comparing sexual self-concept between fertile and infertile women, which was performed after carrying out adjustments for wife’s age, husband's age, duration of marriage, and education of the women, showed that the mean scores of sexual-satisfaction, power-other sexual control, and fear-of-sex subscales were significantly lower in infertile women (P<0.05). There were no significant differences between the fertile and infertile women for other subscales (P>0.05). The comparison of the sexual self-concept subscales between fertile and infertile women is summarized in Table 2

**Table 2:** Comparison of the subscales of sexual self-concept in fertile and infertile women


Subscales of sexual self-concept	Fertile womenn=250	Infertile women n=250	P value^*^

Sexual-anxiety	0.69 ± 0.75	0.80 ± 0.72	0.200
Sexual self-efficacy	2.54 ± 0.68	2.51 ± 0.64	0.741
Sexual-consciousness	2.59 ± 0.66	2.60 ± 0.60	0.896
Motivation to avoid risky sex	3.12 ± 0.65	3.11 ± 0.72	0.245
Chance/luck sexual control	0.71 ± 0.60	0.83 ± 0.61	0.152
Sexual-preoccupation	0.77 ± 0.67	0.84 ± 0.67	0.758
Sexual-assertiveness	1.82 ± 0.55	1.80 ± 0.51	0.523
Sexual-optimism	1.78 ± 0.50	1.82 ± 0.47	0.826
Sexual problem self-blame	1.58 ± 0.78	1.47 ± 0.71	0.065
Sexual-monitoring	1.08 ± 0.62	1.12 ± 0.64	0.684
Sexual-motivation	2.19 ± 0.79	2.31 ± 0.70	0.673
Sexual problem management	2.33 ± 0.69	2.42 ± 0.60	0.386
Sexual-esteem	2.53 ± 0.74	2.54 ± 0.74	0.902
Sexual-satisfaction	2.66 ± 0.84	2.59 ± 0.83	0.033
Power-other sexual control	0.89 ± 0.65	0.77 ± 0.63	0.008
Sexual self-schemata	3.21 ± 0.68	3.42 ± 0.62	0.090
Fear-of-sex	1.67 ± 0.53	1.52 ± 0.60	0.012
Sexual problem prevention	2.90 ± 0.66	2.88 ± 0.71	0.736
Sexual-depression	0.59 ± 0.81	0.61 ± 0.76	0.440
Internal-sexual-control	2.46 ± 0.63	2.46 ± 0.62	0.741


Data are presented as mean ± SD. *; Derived from ANCOVA adjusted for age, husband’s age, duration of marriage, and education as covariates.

## Discussion

The results of this study indicated that some aspects of sexual self-concept were lower among the infertile women as compared to the fertile women. Infertility and the associated sexual problems can cause sexual dysfunction and sexual dissatisfaction (15). Since sexual pleasure is more a product of the mind than of the body, it is very likely that sexual pleasure could be affected by the consequences of infertility such as depression, which in turn interferes with sexual satisfaction (16). Another explanation for such observation is that infertile women appear to be mainly concerned about occurrence of pregnancy during the sexual relationship, and not sexual satisfaction or enjoyment, which can interfere with sexual pleasure among infertile women. Similar findings were reported by other investigators where they reported that sexual dysfunction was lower among infertile women when compared to a sample of fertile couples (17-19).

Since all infertile women in the present study were under treatment and their sexual relationship was mostly based on the schedules of their treatment plan, their sexual activity was mostly planned accordingly and not based on their natural desire. Therefore, the role of the husband in their relationship has been diminished with regard to coercion or control of sexual activities in this group. The results of other studies were also similar. Fear-of-sex was lower in infertile women, which is probably due to the women's high desire to become pregnant. While, in fertile women, fear of unwanted pregnancy might unconsciously lead to fear of sexual relationship. The mean score of this subscale in the other studies was consistent with the present study (3, 20).

The mean scores of sexual-anxiety and sexual-depression in fertile and infertile women were not significantly different. It appears that infertility alone cannot increase sexual-anxiety and sexual-depression in infertile women. Potki et al. (6) found that various aspects of the sexual selfconcept change over a 4-year period. In this evolutionary period, the sexual anxiety of individuals decreases and they feel more sexually friendly so that in the first two years, women's sexual anxiety could be reduced by 70%. Furthermore, as the age and length of the marriage increase, sexual intercourse experiences increase as people feel more comfortable and have more sexual compatibility, resulting in less sexual-anxiety (21). In the present study, the mean duration of marriage was over four years in the fertile and infertile women, which might explain the low level of sexual-anxiety in both groups. Sexual-depression had the lowest score in both fertile and infertile groups, which meant that women had a good acceptance of themselves as a sexual entity and an attractive wife.

There was no significant difference between the fertile and infertile women in terms of sexual self-efficacy and sexual-consciousness. The mean score of these subscales in studies by Hasanzadeh (20) and Zaheri et al. (21) were consistent with those in the present study. Another study, however, showed that sexual self-efficacy in fertile women was higher than that of infertile women (15). Differences in these results can be due to differences in cultural characteristics and the lifestyles of the participants.

There was no significant difference for motivation to avoid high-risk sexual behavior between fertile and infertile women (15). It appears that the high motivation to avoid high risk sex in both groups is probably due to the formation of sexual behaviors within the family framework based on cultural and social values in Iranian context, which in turn lead to the motivation to avoid risky sexual behaviors (22). The low score of selfblame in sexual problems and the high score of sexual problem management and sexual problem prevention in both groups suggest that women do not solely consider themselves responsible for the incidence of sexual problems and dysfunction, but they consider these issues as mutual responsibilities of spouses, and at the same time, have fare management when facing sexual problems.

According to our findings both fertile and infertile groups had low scores in sexual-preoccupation, sexual-assertiveness and sexual-optimism subscales. The low score of sexualpreoccupation suggests that extreme thinking about sex is not a major concern for women. Extreme thoughts about sex will distance people from moderate conditions and may create serious behavioral issues for them. This was not observed in the present study, which might be due to being married and the definition of sexual activities within a family environment. These findings of the present study were in line with the findings of other studies (3, 20). 

The low score of sexual-assertiveness reflects the low level of self-confidence with regards to sexual activities in both groups. According to Bui et al. (23) most women express that they are in a lower rank than men, and they had low sexual-assertiveness and sexual self-efficacy. Sexual assertiveness is not only a way of expressing feelings and communicating about sexual experiences, but it also potentially affects sexual health and satisfaction (24). Sexual optimism is positively correlated with sexual health. Addressing the positive aspects of sexual relationships affects how one perceives his or her sexual future, and this promotes other dimensions of health (25). Given the low scores of sexual-optimism and sexual-assertiveness in both groups and considering the important role of these subscales in sexual health, it is strongly recommended to increase the attention of health professionals to these subscales of sexual self-concept and to provide counseling to women in this regard.

There was no significant difference between fertile and infertile women in terms of sexual-esteem and sexualmotivation. According to other studies, women with an integrated sexual identity are more likely to achieve sexual self-esteem (6). Also high sexual-esteem can be a protective factor against high-risk sexual behaviors (26).

The low score of sexual-monitoring in both groups suggests that the individuals do not pay enough attention to the attitudes and reactions of others about their sexual attitudes and consider sex as a private issue between themselves and their partners. Interestingly, we observed that the importance of privacy in marital sexuality increased the mean score of internal-sexual-control in both groups.

Overall one might argue that there were no significant differences between fertile and infertile women in most measures of sexual self-concept. Two reasons may explain such observations. Firstly, it is possible that sexual selfconcept is an abstract concept and is less associated with reproductive conditions. Secondly, since infertile women usually seek help for their sexual health thus the difference between fertile and infertile women are limited to those aspects that rarely could be dealt with among infertile women. As such, future studies should focus on assessing sexual-satisfaction, power-other sexual control, and fearof-sex among both fertile and infertile women.

Of the strengths of this study was the selection of fertile women from various health centers in Tehran, which included people with various socio-economic backgrounds. Nonetheless, the main limitation of this study relates to its cross sectional design.

## Conclusion

The present study showed that the highest and lowest sexual self-concept scores in both fertile and infertile groups were sexual self-schemata and sexual-depression, respectively. Infertile women scored lower on sexual-satisfaction, powerother sexual control and fear-of-sex compared to fertile women. There was no difference between the two groups in other subscales. Since the low level of sexual satisfaction in infertile women can affect their sexual life and ultimately the quality of their marriage, the enhancement of this subscale of sexual self-concept in infertile women is considered as an important factor in promoting sexual health. To moderated fear-of-sex and power-other sexual control in fertile women, educational-counseling programs can improve the sexual health of women in these subscales. It appears that it is essential to encourage clinicians and providers in infertility and gynecology centers to address the issues of sexual health and sexual problems of both fertile and infertile women and to provide advice on these issues. 
